# Interband Cascade Photonic Integrated Circuits on Native III-V Chip

**DOI:** 10.3390/s21020599

**Published:** 2021-01-16

**Authors:** Jerry R. Meyer, Chul Soo Kim, Mijin Kim, Chadwick L. Canedy, Charles D. Merritt, William W. Bewley, Igor Vurgaftman

**Affiliations:** 1 Naval Research Laboratory, Code 5613, Washington, DC 20375, USA; chulsoo.kim@nrl.navy.mil (C.S.K.); chad.canedy@nrl.navy.mil (C.L.C.); charles.merritt@nrl.navy.mil (C.D.M.); bill.bewley@nrl.navy.mil (W.W.B.); igor.vurgaftman@nrl.navy.mil (I.V.); 2 Jacobs Corporation, Hanover, MD 21076, USA; mijin.kim.ctr@nrl.navy.mil

**Keywords:** interband cascade laser, photonic integrated circuit, chemical sensing, midwave infrared

## Abstract

We describe how a midwave infrared photonic integrated circuit (PIC) that combines lasers, detectors, passive waveguides, and other optical elements may be constructed on the native GaSb substrate of an interband cascade laser (ICL) structure. The active and passive building blocks may be used, for example, to fabricate an on-chip chemical detection system with a passive sensing waveguide that evanescently couples to an ambient sample gas. A variety of highly compact architectures are described, some of which incorporate both the sensing waveguide and detector into a laser cavity defined by two high-reflectivity cleaved facets. We also describe an edge-emitting laser configuration that optimizes stability by minimizing parasitic feedback from external optical elements, and which can potentially operate with lower drive power than any mid-IR laser now available. While ICL-based PICs processed on GaSb serve to illustrate the various configurations, many of the proposed concepts apply equally to quantum-cascade-laser (QCL)-based PICs processed on InP, and PICs that integrate III-V lasers and detectors on silicon. With mature processing, it should become possible to mass-produce hundreds of individual PICs on the same chip which, when singulated, will realize chemical sensing by an extremely compact and inexpensive package.

## 1. Introduction

Recent years have seen an accelerating development of optical technologies operating in the midwave infrared (mid-IR). This has been driven partly by the opportunities for sensitive spectroscopic detection of trace chemicals [1], since in gas form the narrow mid-IR absorption lines that can positively identify a given chemical are 2–3 orders of magnitude stronger than those in the near IR. Applications include monitoring greenhouse gases [1], industrial process control [2], combustion diagnostics [3], clinical breath analysis [4], and isotope differentiation [5]. However, the long-term market for mid-IR chemical sensing products will depend in large part on such practical factors as cost, power budget, and system footprint. While other sensing techniques, such as gas chromatography and monitoring variations in the properties of metal oxide semiconductors or polymers, may also be employed [6], laser spectroscopy combines the advantages of selectivity, sensitivity, stability, longevity, and range in the case of remote sensing [5].

It follows that rather than using bulky and costly discrete optical elements interacting across free space, the ideal spectroscopic sensing system will occupy a single semiconductor chip that is inexpensive to produce in mass quantities [5]. This will require the incorporation of at least one mid-IR source, optical sensing path, and detector, along with interconnections linking them, into a single photonic integrated circuit (PIC). However, the progress so far toward this objective has been incremental. While mid-IR lasers and detectors have been combined [7,8,9], and sensing waveguides have been integrated with detectors [10,11], we are aware of only one report to date of all three fundamental components being integrated on the same mid-IR PIC. This was a QCL and QCD integrated on the same chip with a suspended membrane waveguide patterned with subwavelength slots, which was designed for sensing that had not yet been demonstrated [12]. Although still immature, more complete sensing PICs operating in the near IR have been reported [13].

In this work, we lay out a comprehensive and practical framework for combining all the optical elements required to integrate a high-performance mid-IR chemical sensor on a PIC [14]. The platform is an interband cascade laser (ICL) structure [15,16,17,18] residing on its native GaSb substrate. The ICL combines a relatively long upper-level lifetime, characteristic of semiconductor interband transitions, with the voltage-efficient cascading scheme originally introduced for the quantum cascade laser (QCL, which employs inter-subband transitions to produce light) [19]. Both electrons and holes are present in each stage of the ICL’s cascaded active region, even though the contacts inject and remove only electrons. The ICL has become the leading coherent optical source for many mid-IR applications between 3 and 6 μm [18]. It operates with drive power as low as 29 mW [20] and wallplug efficiency as high as 18% [21] for continuous wave (cw) operation at room temperature. However, it is less mature than the QCL, and a wealth of complex physics responsible for various aspects of the ICL operation have yet to be fully unraveled.

In the following sections, we describe how building blocks, corresponding to the individual optical components, can be used to construct a variety of highly compact chemical sensing architectures [14] that are suitable for fabrication in a standard cleanroom. Each combines one or more ICLs with interband cascade detectors (ICDs), passive waveguides, and/or other active and passive elements. We also describe an edge-emitting laser configuration that optimizes stability by minimizing parasitic external optical feedback, besides having the potential to operate with lower drive power than any mid-IR laser now available. Most of the configurations described below have not been described previously in the open literature.

While integration on the native III-V chip will be assumed in most of what follows, many of the same configurations may also be advantageous when integration is on a silicon chip. The University of California Santa Barbara (UCSB), NRL, and University of Wisconsin recently reported the first successful integration of mid-IR QCLs on silicon [22,23]. Those PICs were formed by heterogeneously bonding active III-V wafer materials to a silicon-nitride-on-insulator (SONOI) chip that was pre-patterned with passive waveguides and DFB gratings. Laser ridges were then processed from the back. However, the performance thus far has been limited by an inefficient transfer of laser power from the hybrid III-V/silicon waveguide of the gain region to a passive silicon-based waveguide that can couple the laser beam to other optical elements on the PIC. UCSB and NRL subsequently reported the integration of ICLs on silicon using an analogous heterogeneous bonding approach [24], although inefficient coupling between the hybrid and silicon-based waveguides again limited the output power. An alternative approach to the integration of mid-IR sources on silicon has been to grow InP-based QCLs directly onto silicon substrates [25,26].

## 2. Building Blocks: The Laser, Detector, and Passive Waveguides, Plus Other Active and Passive Optical Components

**Figure 1:**
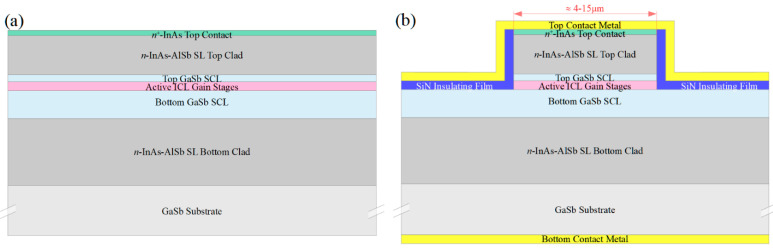
(**a**) Schematic layering of an interband cascade laser (ICL) grown on GaSb. The primary regions are the active gain stages, top and bottom high-index/low-loss *n*^−^-GaSb separate confinement layers, top and bottom InAs-AlSb superlattice optical cladding layers, and an *n*^+^-InAs top contacting layer. (**b**) Schematic cross-section of an ICL narrow ridge waveguide formed by etching through the active stages of the wafer material illustrated in Figure 1a. The same ridge structure may provide gain when forward biased, or function as a detector under zero or reverse bias. Typical layer thicknesses are 2–4 µm for the bottom cladding layer, 500–900 nm for the bottom SCLs, 200–400 nm for 5–10 active gain stages, 100–300 nm for the top separate confinement layer (SCL), 1.2–2 µm for the top cladding layer, and 20 nm for the top contact layer.

We will assume the starting material to be the epitaxial layers corresponding to an ICL grown on GaSb, which are illustrated schematically in Figure 1a. Here the active gain stages are surrounded by lightly doped *n*-type GaSb separate confinement layers (SCLs), which are in turn surrounded by InAs-AlSb superlattice (SL) optical cladding layers. The GaSb substrate and buffer layer are *n*^+^-doped to allow for bottom contacting to the substrate, and the epitaxial layers are capped by an *n*^+^-InAs top contacting layer. While a standard off-the-shelf ICL structure may be suitable, it is preferable to modify the design slightly by making the bottom SCL somewhat thicker and the top SCL somewhat thinner (or eliminating it altogether), as will be discussed below. We also note that analogous QCL structures may be employed in most of the configurations discussed below, with *n*^−^-InGaAs substituted for the GaSb SCLs, InP for the InAs-AlSb SL cladding layers, and *n*^+^-InGaAs for the top contacting layer. 

Figure 1b schematically illustrates a narrow ridge waveguide structure processed from the epitaxial material shown in Figure 1a. The etch that defines the ridge generally proceeds to below the active stages, to prevent the severe lateral current spreading that would otherwise occur in either an ICL [27] or QCL [28]. A dielectric, such as SiN, is deposited on the ridge sidewalls, followed by metallization to provide a top electrical contact. The bottom contact is usually made to the *n*^+^-GaSb substrate, although it is also possible to fabricate two top contacts [24]. A substantial fraction of the optical mode in a conventional ICL resides in the top and bottom *n*^−^-GaSb SCLs, which exhibit low loss and high refractive index. All of the active ridges, as well as the passive waveguides discussed below, should be ≈ 4–15 µm wide to assure lasing and propagation in a single lateral mode. 

The ridge illustrated in Figure 1b can provide gain when a forward bias is applied, and lasing if the ridge is incorporated into a cavity. However, the same narrow ridge can function as an interband cascade light emitting device (ICLED) if no cavity provides feedback and the emitter operates at a forward bias that corresponds to net propagation loss. At higher biases that induce net gain (optical gain minus internal loss), it will function as an interband cascade amplified spontaneous emitter (ICASE), so long as the feedback remains insufficient to induce lasing.

It has also been demonstrated that, under zero or reverse bias, the layering structure of Figure 1a, and therefore the narrow ridge waveguide of Figure 1b, can operate as an interband cascade detector [29]. In an ICL operating under forward bias, the electron injector with chirped quantum well thicknesses transfers electrons from the semimetallic interface that separates the electron and hole injectors to the active electron quantum wells, where they recombine with holes in the active hole quantum well [20]. In an ICD, on the other hand, electrons photoexcited in the active quantum wells of a given stage produce photocurrent in the opposite direction, by flowing downhill through the electron injector toward the semimetallic interface, where they recombine with photoexcited holes from the next stage. Thus, whereas a single electron injected electrically into an *N*-stage ICL can emit *N* photons, an ICD must absorb *N* photons to transfer a single electron across all the stages to provide a photocurrent. ICDs displayed high detectivity at room temperature [29,30,31]. 

U. Oklahoma recently reported an ICL and ICD integrated on the same native GaSb chip [8], although separated by an air gap rather than a passive waveguide as will be required for versatile integration on a PIC. Other groups have also demonstrated ICLs and ICDs processed from the same wafer material, although not coupled on the same chip [32,33]. The ICD’s cut-off wavelength is always roughly equal to the ICL emission wavelength, since both are determined by the bandgap of the active quantum wells. This contrasts the less ideal alignment for a QCD processed in parallel with a QCL. In that case, an “extraction” sub-band lies about one optical phonon energy (*ħω*_o_) below the lower lasing level to provide rapid depopulation following a stimulated emission event. At zero bias, most electrons populate the extraction sub-band, so the QCD’s absorption peak occurs closer to *ħω* + *ħω*_o_ than to the lasing energy *ħω*. Nonetheless, specially designed QCLs residing on the same native InP chip [8] with QCDs have produced up to 1 W of cw emission at 15 °C [34]. 

**Figure 2:**
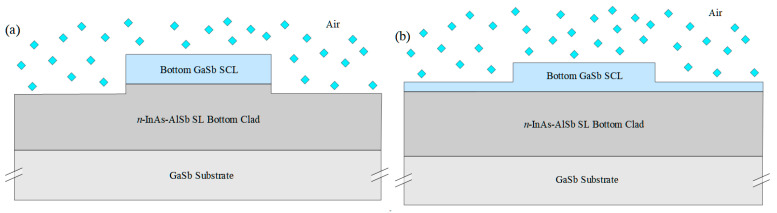
Schematic cross sections of passive waveguides processed from the ICL epitaxial structure of Figure 1a. The ridge is formed by etching away the top contacting layer, top clad layer, top SCL, and the active stages, to leave the bottom SCL that serves as the waveguide core and the bottom InAs-AlSb SL that forms the bottom clad. Outside the narrow ridge, either the bottom SCL is fully etched away, with the etch stopping near the top of the bottom cladding layer (**a**), or the etch stops within the bottom SCL (**b**). If the tops and sides of these passive waveguides are left exposed, as shown in the figures, propagating light will evanescently couple to an ambient sample gas or liquid. A typical overlap of the optical mode profile with the ambient gas will be illustrated below.

A chemical sensing PIC will also need low-loss passive waveguides, to connect the other components and in some approaches to provide evanescent coupling to an ambient sample gas or liquid that may or may not contain an analyte of interest. Figure 2a,b illustrate two passive waveguide structures that may be processed from the ICL material of Figure 1a. They are realized by etching away the heavily doped top contacting layer, top cladding layer, top SCL, and active gain stages, with the etch stopping near the top of the bottom SCL. Whereas typically the top and bottom SCL thicknesses in a conventional ICL are equal, in this case the structure should be redesigned such that the bottom SCL is thicker and the top SCL thinner, as shown in Figure 1a, or absent altogether. In exchange for a modest reduction in optical confinement in the active gain stages, this will thicken the high-index core of the passive waveguide, and better align the active and passive waveguide modes along the vertical axis. For example, if a conventional ICL structure emitting at 3.4 µm, with seven stages and SCL thicknesses of 700 nm on both top and bottom, is redesigned, such that the top SCL is thinned to 450 nm while the bottom SCL is thickened to 950 nm, the optical confinement factor in the active region decreases from 15.7% to 10.2%. However, in exchange, 80% of the fundamental mode then lies below the active core, a large fraction of which should couple to the passive waveguide following removal of the active core to form a passive waveguide. An interface between active and passive waveguides will be illustrated below.

In Figure 2a, the bottom SCL is etched away entirely outside the ridge, with the etch stopping near the top of the superlattice bottom cladding layer. Alternatively, Figure 2b shows the etch outside the ridge, stopping within the bottom SCL. In both cases, the beam propagates in the ridge waveguide formed by the bottom *n*^−^-GaSb SCL as its core and the bottom InAs-AlSb SL as its bottom clad. The top and sides of the ridge may be encapsulated in a dielectric or left exposed for evanescent coupling to ambient. 

The optical loss in these passive waveguides should be low when the contributions by scattering and the other optional treatments described below are minimized. Therefore, a relatively long sensing waveguide that maximizes net coupling to the sample gas may be feasible (>>1 cm^−1^). The path geometry may be straight, or follow a meandering pattern, as shown in some of the figures below. Or it may be configured as a ring (see below) or other resonator configuration, to extend the effective path length and also provide spectral selectivity imposed by the resonance. 

Nonetheless, the modal overlap with ambient is relatively weak because the uppermost GaSb layer of the waveguides shown in Figure 2a,b has a high refractive index, while the top cladding layer is the air containing the analyte. For this reason, it may beneficial to pattern a slot waveguide, sub-wavelength grating, or other nanoscale structure that increases the coupling of the beam to ambient, employ a hollow-core waveguide that provides propagation through free space, or coat the top and sides of the waveguide with a chemical sorbent that selectively concentrates a given class of chemicals. Those options will be discussed briefly in Section 6 below, although a detailed analysis falls outside the main scope of this work. 

The lasers, detectors, and passive waveguides described above can also be integrated into more complex PICs that incorporate other optical components for enhanced capability. For example, an arrayed waveguide grating (AWG) can spectrally combine the beams from an array of distributed feedback (DFB) [35,36] or distributed Bragg reflector (DBR) lasers, with slightly different grating pitches, into a single output for use elsewhere on or off the chip. Or spectroscopy can be performed by using an AWG to separate a single broadband input into spectral components that are detected individually [37]. 

We must also consider how to couple the different types of waveguides for optimal beam transfer from one active or passive optical component to another. For example, one end of a laser cavity employing the narrow ridge waveguide illustrated in Figure 1b may be defined by simple butt coupling to a passive waveguide (Figure 2). Reflection for feedback is then provided by the differing mode profiles, as well as the different modal indices of the two waveguides. Of course, this approach provides relatively little opportunity for tuning the reflectivity of the output mirror. It is also worth noting that, since the active waveguide shown in Figure 1a is taller than the passive waveguides of Figure 2, the centroid of its mode profile along the vertical axis is higher than in the passive waveguides. Therefore, to minimize transmission into the ambient above the passive waveguide, the upper portion of the active waveguide at the interface should be coated with a dielectric and gold for high reflection (HR). This is illustrated below. It may also be desirable to taper the passive waveguide to a width somewhat narrower than the active laser waveguide since a ridge narrow enough to preclude lasing in higher-order lateral modes may nonetheless allow for propagation in multiple modes when no gain is present. 

**Figure 3:**
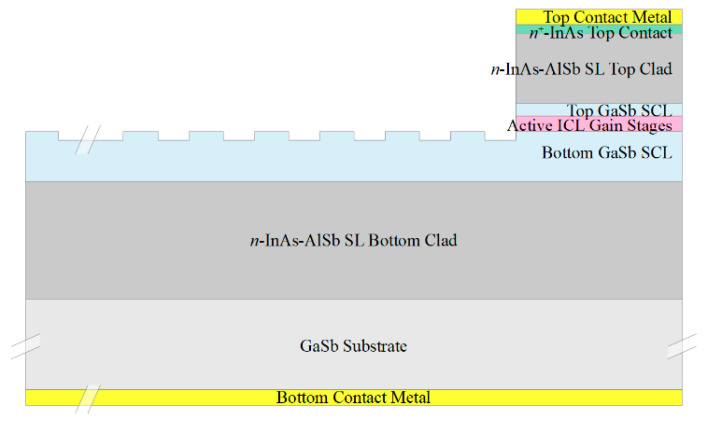
Schematic of a third-order distributed Bragg reflector (DBR) mirror that defines one end of an ICL cavity. If the DBR is designed for outcoupling the laser light, the mirror couples into a passive waveguide positioned farther to the left in the figure.

In principle, the laser output coupling is more controllable if a distributed Bragg reflector (DBR) is instead processed at the interface between the active and passive waveguides (and possibly at the other end of the cavity to define the back mirror as well) [38]. When a III-V laser is bonded to silicon to form the PIC, it is relatively straightforward to pre-pattern a DBR grating into the silicon before the active gain material is bonded and processed [23]. However, for ICL-based PICs residing on the native GaSb substrate, the DBR must be etched into the ICL wafer material, as illustrated in Figure 3. For a DBR, defining one end of the laser cavity (or detector absorber region) that couples to a passive waveguide, it is preferable to etch the grating into the passive waveguide (at left in the figure), rather than into the full layered structure (at right). Given the spatial resolution and sidewall angle that can be achieved routinely for a given grating period when GaSb-based laser structures are processed with reactive ion etching, the processing yield is likely to be higher if a third-order rather than first-order grating is employed. For the example of an ICL emitting at 3.5 µm, the period for a third-order grating with 50% duty cycle is roughly 1.5 µm. The reflectivity can then be tuned to a desired value by varying the grating length and/or etch depth. For maximum reflection by a mirror that is not intended for output, our simulations indicate that a grating length of at least 100 µm is preferred, which, with an etch depth of 280 nm, would produce ≈ 90% reflectivity. The corresponding parameters for a first-order grating are a 500 nm period and 120 nm etch depth.

The waveguide for an in-plane ICD may also be bound on both ends by DBRs, or on one side by a DBR and on the other by a cleaved or etched HR-coated facet, to form an in-plane resonant cavity detector [39,40]. By taking advantage of multiple in-plane passes through the absorber, a shorter ICD waveguide that generates lower dark current can maintain high quantum efficiency for enhanced specific detectivity D*. 

## 3. Sensing within the Laser Cavity

Some or all of the active and passive optical elements described above can be combined in a number of ways to form an on-chip chemical sensor. One of the simplest is to imbed an active or passive sensing waveguide within the laser cavity.

**Figure 4:**
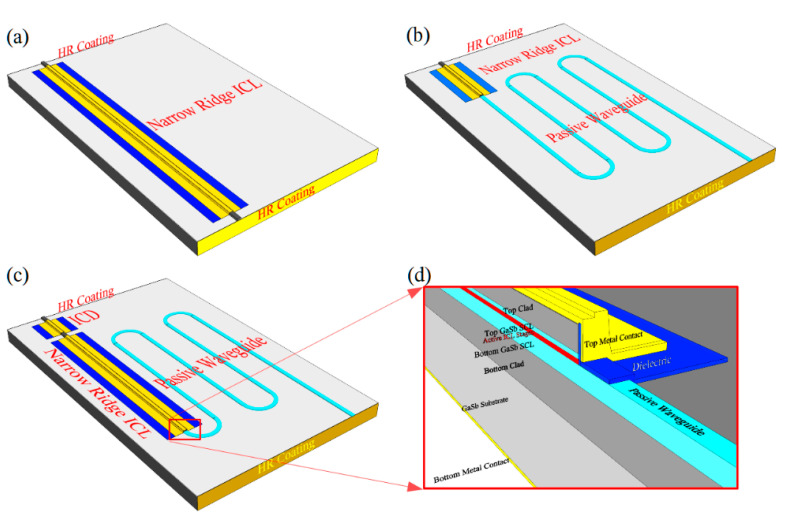
Schematics of laser cavities defined by two HR-coated mirrors: (**a**) the laser does not interact with any optical element outside its own cavity; (**b**) a passive waveguide inside the laser cavity evanescently couples to ambient; (**c**) both a passive sensing waveguide and an interband cascade detector (ICD) are incorporated inside the cavity; (**d**) detail of the interface between the active and passive waveguides.

We begin with the simple baseline configuration, illustrated in Figure 4a, which is formed by placing HR-coated cleaved facets at both ends of a narrow ridge, although one or both ends of the cavity may just as well be bound by an etched facet or high-reflectivity DBR. Since there is no opportunity for feedback from an external optical element to influence the laser operation, its stability should be limited only by the drive electronics and environmental temperature fluctuations (typically taking place on a relatively long-time scale). Ordinary vibrations should have little effect, since all of the fixed internal lengths will vibrate in unison. Very low drive power is also possible in principle, as will be discussed further below.

**Figure 5:**
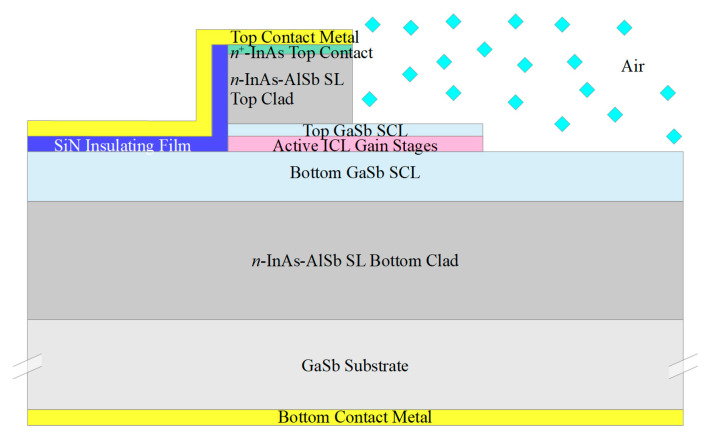
Schematic cross-sectional profile of a waveguide with part of the top SCL and one sidewall of the narrow ridge left exposed to ambient to allow evanescent coupling of the lasing mode to a sample gas or liquid.

Of course, a laser having no interaction at all with anything outside its own cavity cannot perform a useful function. However, we can provide interaction without introducing any external optical feedback by configuring the ridge geometry to impose finite overlap of the propagating optical mode with ambient. One conceptually simple approach is to modify the ridge waveguide profile from Figure 1b such that part of the top SCL is exposed, as illustrated schematically in Figure 5. Spectral selectivity may also be imposed by patterning a DFB grating into the partially-exposed ridge. 

**Figure 6:**
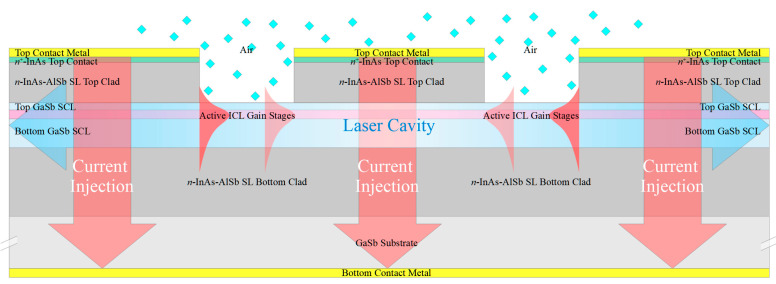
Schematic side view of an active gain waveguide in which notches are etched to allow overlap of the propagating optical mode with ambient.

An alternative possibility is to open one or more notches in the top of some portion of the active gain waveguide, as shown schematically in Figure 6, such that the propagating mode evanescently couples to ambient. Each notch should be narrow enough (<200–300 μm) to allow for current spreading and self-pumping to inject carriers into its active stages.

A third possibility is to longitudinally divide the cavity into active and sensing sections, as shown schematically in Figure 4b. The active waveguide section includes the full ICL structure containing the active gain stages, as in Figure 1b, whereas the sensing section comprises a passive waveguide with exposed top and side surfaces, such as those in Figure 2. The sensing section may be relatively long, (e.g., >1 cm) to enhance the chemical detection sensitivity, since the passive waveguide loss should be low and absorption by the sample gas is weak in most cases. The passive sensing waveguide may be straight, leading directly to a mirror that defines one end of the cavity, or it may follow a long meandering path (with bend radius large enough to result in little additional loss), as shown in Figure 4b. 

Assuming that the amplitude and spectral characteristics of the beam propagating in the cavity are modified by the presence and concentration of a chemical species of interest in the ambient sample gas, we still need a means for quantifying the effect. While a detector can be incorporated, as will be discussed below, it is not required. Instead, we can monitor the compliance voltage in the laser’s *I–V* characteristics when a constant current is injected [41]. When the cavity loss increases, due to absorption by the sample gas, the lasing threshold increases and the radiative recombination rate decreases at a given current injection level above threshold. Therefore, a higher voltage must be applied to maintain the same current. Phillips et al. observed a 0.15 V increase in the compliance voltage for a QCL emitting near 7.7 µm when the laser wavelength swept across an absorption line of water vapor [41]. In that experiment, the sample gas resided in an external portion of the laser cavity (which coupled to a grating for tuning the lasing wavelength), rather than coupling evanescently to the beam propagating in a waveguide. Without an external cavity, the wavelength of the laser illustrated in Figure 4b can be tuned across the absorption resonance by current or temperature, and the dependence of compliance voltage on current, then compared to that observed when the chemical species of interest is not present in the ambient sample gas. Multiple DFB or DBR lasers on the same chip, which all incorporate sensing waveguides, can provide a reference by tuning to different wavelengths. 

A further alternative is to monitor the absorption resulting from evanescent coupling to the sample gas by incorporating an additional interband cascade detector waveguide section into the ICL cavity, since the same ridge waveguide is suitable for both. For the example shown schematically in Figure 4c, the ICD is placed on one side of the active gain section and the passive sensing waveguide on the other. However, the detector may also be placed inside the laser cavity when evanescent coupling to the sample gas occurs within the active gain section; for example, using the partially-exposed waveguides shown in Figure 5 and Figure 6, rather than in a separate sensing waveguide. The ICD and ICL sections must naturally be contacted individually, with a gap in between to prevent crosstalk. The detector section must be short enough that its absorption of lasing photons does not dramatically degrade the laser performance. However, high sensitivity should be achievable with a very short detector because the in-plane absorption is quite strong. 

## 4. Integrated ICL with Enhanced Stability

Trace chemicals can be detected by measuring the differential absorption induced by a constituent of the sample gas, against the background when the species of interest is absent. It follows that the minimum detectable absorption depends critically on the stability of the laser, since any jitter or other fluctuation will easily wash out a small differential signal. 

A major cause of instability is feedback from a secondary optical cavity that forms due to reflection from one or more surfaces encountered outside the intended primary cavity [42,43,44,45]. For example, reflection may occur at an external optic or other surface encountered after the beam leaves the output facet of an edge-emitting laser. One study found that as little as −30 dB feedback could broaden the spectrum and degrade the relative intensity noise (RIN) of an edge-emitting DFB laser [46]. Furthermore, inevitable mechanical and thermal vibrations cause the secondary cavity length to vary, thereby inducing temporal “jitter” of the magnitude and spectral characteristics of the parasitic modes. A conventional edge-emitting diode laser with cleaved output facet that is either AR coated (reflectivity *R* ≤ 2%) or uncoated (typically *R* ≈ 25–40%) [21] is especially susceptible to unwanted feedback, since a significant fraction of the light returned to the facet following external reflection is transmitted back into the cavity, and even a modest amount of feedback can degrade the laser stability. While an optical isolator can minimize the external feedback, they are too bulky (as well as expensive and wavelength-specific) for incorporation into an ultra-compact chemical sensing system. 

An advantage of PIC-based sensors that place the sensing waveguide entirely within the laser cavity is the potential to minimize nearly all parasitic feedback by more effectively isolating the laser cavity on the chip. Given an ultra-low-noise electrical driver, the lasers illustrated in Figure 4a–c should be extremely stable because virtually all feedback from external optical elements is eliminated. While external light can, in principle, be injected into the laser cavity via ridge processing imperfections or even interactions with the analyte, those sources should be entirely negligible compared to the nearly inevitable feedback, due to external reflection of light emitted at a facet back into the cavity in the absence of an optical isolator. On the other hand, the more general flexibility of this laser design for incorporation into a PIC, intended for a function extending beyond intracavity chemical sensing, is severely limited by the absence of an output beam. 

In practice, we can trade output power from the on-chip laser against stability by adjusting the fraction of light coupled out during each pass through the laser cavity. For example, when relatively little power is needed, we can increase the reflectivity of the output facet, say, to ≈ 90%. Low mirror loss provides the further advantage of lower threshold current and drive power, especially if the cavity length is also shortened as will be discussed further. In principle, the most straightforward approach to increasing the reflectivity at a facet, while still allowing output, is to deposit a multi-layer Bragg dielectric coating. However, such coatings are typically expensive and challenging to process, especially at longer wavelengths, where each layer becomes proportionally thicker. Alternatively, the reflectivity of a DBR end mirror that couples to a passive waveguide can be adjusted for optimal trade-off between laser stability and output power. 

**Figure 7:**
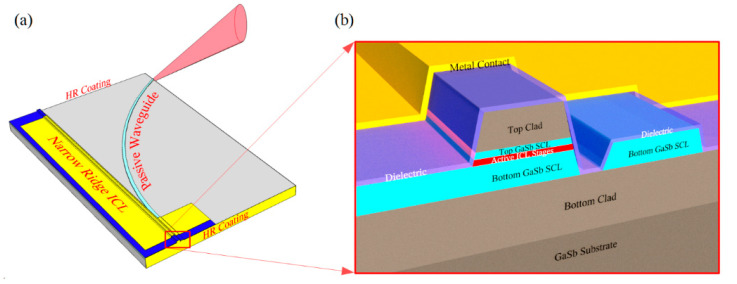
(**a**) Schematic showing light extraction from a laser cavity via evanescent coupling to a passive waveguide that runs parallel to a section of the active gain waveguide; (**b**) detailed cross-section of the two waveguides at the HR-coated facet.

A more flexible way to tune the laser output per pass through the cavity is to evanescently out-couple the light laterally, to a second (passive) waveguide that runs parallel to the active gain waveguide over some section of the cavity [14]. Figure 7 illustrates that once a desired fraction of the beam has coupled into the passive waveguide, it can bend away from the primary waveguide and proceed either toward a facet for emission from the chip (as shown in the figure), or elsewhere on the chip for functionality within the PIC. In the former case, the facet at which light is emitted may be AR coated, and the passive waveguide may intersect at an angle to further minimize the potential for reflection back into the laser cavity. Alternatively, the output waveguide may retain the full laser structure with top contact, which is then forward biased for amplification of the output signal. 

The space between the two coupled waveguides should be filled with a low-loss dielectric, such as SiN, and the top of the passive waveguide’s coupling section covered by a dielectric to minimize optical loss due to modal overlap with the laser’s top contact metal. The coupling strength between the active and output waveguides may be tuned broadly by varying their separation distance (e.g., ≈ 0.3 to 2 µm), the etch depth in the region separating them, and/or the length over which they run in parallel. Simulations for an ICL emitting at λ = 3.5 µm indicate that if both waveguides are 5 µm wide, are separated by 500 nm, and the coupling length is 19 µm with no etch of the region separating them, ≈ 27% of the light transfers per pass from the active to the passive waveguide [14].

**Figure 8:**
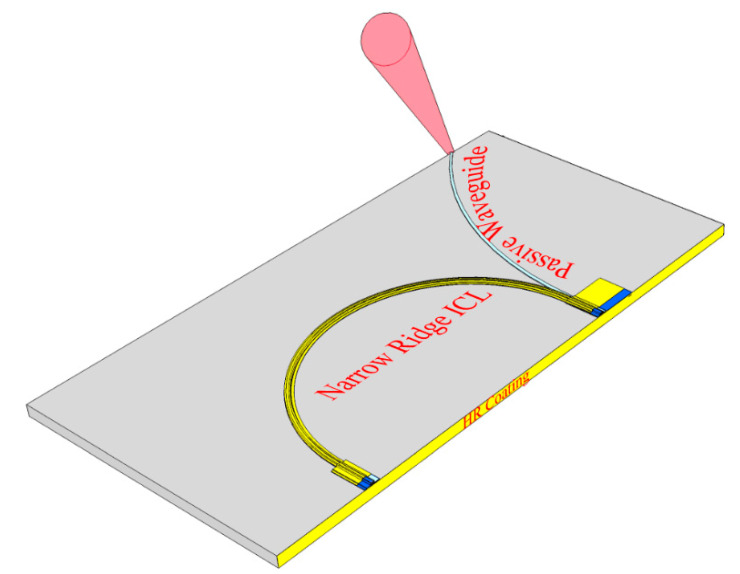
Schematic of a curved laser cavity with laterally coupled output and both end mirrors provided by the same HR-coated facet.

Lateral out-coupling can provide light for the PIC or external emission from a laser whose cavity is terminated on both ends by cleaved HR-coated facets, as shown in Figure 7. Alternatively, the cavity can curve, as shown in Figure 8, such that a single HR-coated cleaved facet provides both end mirrors. 

We emphasize that the threshold drive power for a laser with any of the cavity designs, shown in Figure 7 and Figure 8 (or Figure 4), can be extremely small, even lower than the record mid-IR value of 29 mW already reported for an ICL [20]. With minimal mirror loss, because the cavity is defined by two HR-coated facets, *L*_cav_ can be shortened beyond the usual limit of ≈ 0.5 mm. While the minimum practical length that a thinned GaSb-based device structure can be cleaved with high yield using conventional methods is also ≈ 0.5 mm (potentially shorter if at least one end mirror is an etched facet or DBR), the configuration in Figure 8 makes a shorter length straightforward, with an ultimate limit imposed by bending loss. If the active gain length is reduced only modestly to 0.3 mm, along with a ridge width of 4 µm, current density (slightly above threshold) 250 A/cm^2^, and threshold voltage 3 V, with a power budget of <10 mW per laser, is quite realistic [14]. Such low drive power can easily be supplied by a small solar cell. A further advantage of the very short cavity is that with greater spacing between the longitudinal optical modes, emission in a single mode may become possible without any need to pattern a DFB grating.

**Figure 9:**
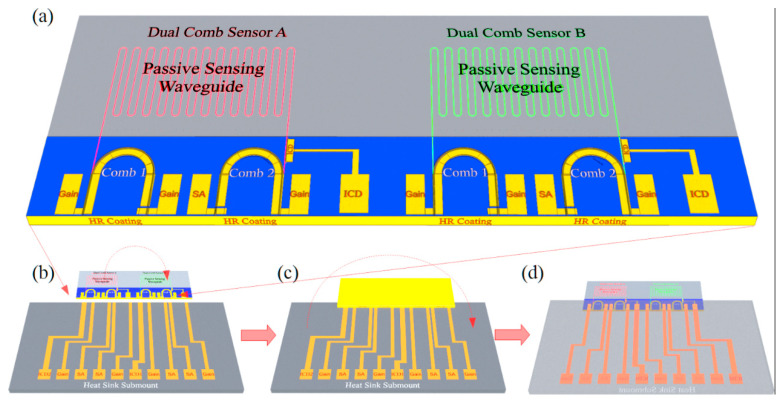
(**a**) Schematic of a PIC that integrates two dual comb spectrometers with multiple ICLs and ICDs grouped at one end and multiple sensing waveguides at opposite end. This allows the lasers to be electro-plated with gold and mounted epitaxial-side-down on a patterned heat sink submount, while the sensing waveguides hang over the edge of the submount to provide exposure to the ambient sample gas; (**b**) The chip and patterned submount before flip-chip bonding (**c**) The chip and submount after flip-chip bonding; (**d**) The reverse side of the flip-chip-bonded PIC, showing the exposed sensing waveguides.

Terminating both ends of the laser cavity at the same HR-coated facet may be especially beneficial in PICs that integrate multiple lasers, which are mounted epitaxial-side-down for maximum heat dissipation, on the same chip with multiple passive sensing waveguides that must be exposed to ambient. Figure 9 illustrates that it can be straightforward to design the PIC such that the lasers are grouped at one end for flip-chip bonding to a thermal submount, while the sensing waveguides are grouped at the other end that hangs over the edge of the submount for exposure to ambient. We also note, however, that an ICL’s threshold input power density is low enough that it can generally operate cw at room temperature even when mounted epi-up, only with lower maximum output power than for epi-down mounting. 

## 5. Integration of the Optical Components on a Sensing PIC

**Figure 10:**
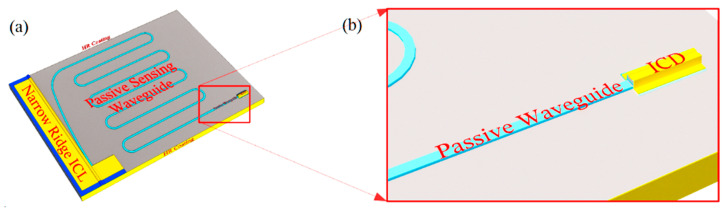
(**a**) Schematic of an ICL with lateral out-coupling, extended passive sensing waveguide, and interband cascade detector integrated on the same III-V chip; (**b**) detail of the passive waveguide leading to the ICD.

Once the laser output has coupled to a passive waveguide, which may have an air top cladding, as in Figure 4a,b for evanescent coupling to an ambient sample gas, absorption spectroscopy can be implemented to sense the presence of analyte molecules. The other end of the passive waveguide can then couple to an ICD that resides on the same chip, as illustrated in Figure 10. The optimal waveguide length that maximizes detection sensitivity depends on such factors as absorption strength by the analyte, parasitic loss in the passive waveguide, and of course laser stability. Note also that, instead of using current or temperature to scan the laser’s emission wavelength across an analyte absorption feature, the same PIC may integrate multiple sensors operating at both fingerprint absorption feature and reference wavelengths. 

**Figure 11:**
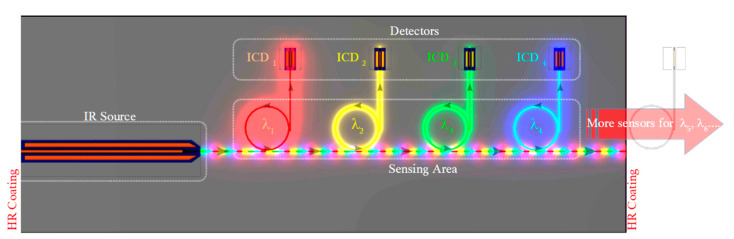
Schematic of an on-chip chemical sensor in which an ICL source emits into to a passive bus waveguide for coupling to a series of ring resonators [37]. Each ring extracts a different comb of resonance wavelengths. The individual rings then evanescently couple to ICDs that quantify the spectral dependence of the absorption.

Instead of a simple passive waveguide that may follow a straight or meandering path, the sensing waveguide may also take the form of a high-Q resonator (with exposed top surface) that allows many passes of the beam. Figure 11 illustrates that a single ICL may emit into a passive bus waveguide that couples to a series of ring resonators [40]. Each ring in the series selectively extracts its own comb of resonance wavelengths from the bus, to determine the spectral dependence of the absorption. Each ring in turn couples to an ICD that quantifies the signal. Coupling to the resonators may be increased by shaping the rings as racetracks rather than circles.

We have found that for ICLs processed on the native GaSb substrate, the bending loss may be too large to maintain high *Q* if the ring diameter is substantially less than 100 µm. This diameter corresponds to a free spectral range (FSR) between resonances of ≈ 2.5 cm^−1^, meaning that one ring at a time could select a narrow emission line from a temperature- or current-tuned DFB laser, but could not select a single narrow range of wavelengths from a broadband source such as an ICLED. Since the bending loss from sidewall scattering would be lower for a PIC processed on silicon rather than GaSb, a silicon ring could have a smaller diameter and larger FSR.

Alternatively, a passive waveguide section within a laser cavity, e.g., defined on both ends by HR-coated cleaved facets as in Figure 4b, may couple to a single ring (or two rings with slightly different diameters), which in turn couples evanescently to a separate passive waveguide that leads to an ICD. While the laser may employ a DFB or DBR grating to induce lasing in a single mode, in which case the ring resonance must be tuned to coincide with the laser wavelength, this is generally unnecessary because, with proper design, the double ring resonance will select a single longitudinal lasing mode [47]. The ring’s resonance wavelength can then be tuned with local temperature. 

Recent reports of ICL frequency combs [32,33,48] have demonstrated their potential for sensitive chemical detection. While the physics of ICL combs is not yet well understood, and in many cases the comb linewidth is not sufficiently narrow for practical applications [49,50], we expect these issues to be resolved by research aimed at systematically minimizing the group velocity dispersion and optimizing the device structure for comb operation. Dual-comb spectroscopy offers a combination of high spectral resolution over a broad spectral bandwidth with very short acquisition time on the order of milliseconds [33,51]. A fast detector observes the multi-heterodyne beating of two combs with slightly different teeth spacings (determined by the cavity lengths), from which the IR laser spectrum is mapped into the RF frequency domain where it is more easily measured. The cavities of passively mode-locked ICL combs are subdivided into individually contacted gain and saturable absorber (SA) sections, which are separated by a non-contacted gap. This represents a straightforward modification of the cavity geometries illustrated in Figure 7 and Figure 8. 

**Figure 12:**
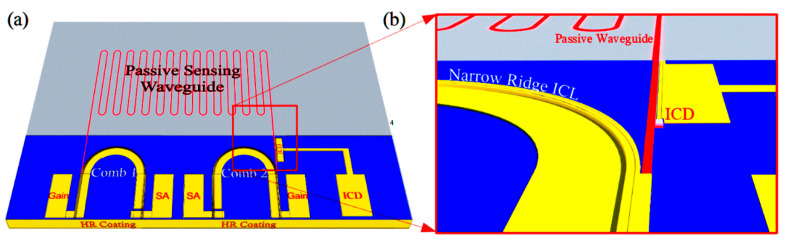
(**a**) On-chip dual-comb spectrometer, in which both facets of both frequency comb ICLs terminate at the same HR-coated facet. Lateral output from the first comb couples to an extended passive sensing waveguide before input to the ICD, while output from the local oscillator comb transfers directly to the same ICD; (**b**) detail showing both inputs coupling evanescently to opposite ends of the ICD.

Figure 12a illustrates that a fully-integrated on-chip dual-comb spectrometer can be realized by integrating two ICL frequency combs (in which the spacing may be adjusted by tuning the pump current in the gain section as well as reverse bias on the SA section) on the same PIC with an extended passive sensing waveguide and an ICD with GHz cut-off frequency. The beam from one comb interrogates the sample gas before detection by the high-speed ICD. The other comb provides the local oscillator (LO) reference beam, which proceeds directly to the ICD via a passive waveguide so short that, even without encapsulation, the sample gas has negligible effect on its power input to the detector. An RF spectrum analyzer then receives the combined signals. In order to resolve the RF heterodyne beating, it is fortunate that ICDs with active areas small enough to minimize capacitance have displayed 3-dB bandwidths >> 1 GHz [31,33]. Because each stage of the ICD is thin enough to make the transit time quite short, a reduction in the RC time constant is sufficient for this purpose. Of course, laser stability also plays a critical role in the detection sensitivity [33]. 

Figure 12b illustrates that the two inputs to the ICD may evanescently couple laterally from opposite ends, although both inputs may alternatively combine at a Y-Junction, which is then inputted to the ICD. The passive waveguide sections running parallel to the ICD should be long enough for high QE and terminated in a manner that minimizes unwanted reflections back into the lasers. Because the lasers in the figure have both end mirrors terminating at the same HR-coated facet, both can occupy the same end of the chip as the ICD while the passive sensing waveguide resides at the other end. Therefore, both frequency comb ICLs can be mounted epitaxial side down while the sensing waveguide hangs over the edge of the mount to allow evanescent coupling of its top surface to the ambient sample gas (as in Figure 9). It is also worth noting that this cannot be accomplished using DBRs to terminate the cavities at the output ends of the two lasers, since the comb bandwidth would then be limited to the DBR stopband.

The on-chip dual-comb spectrometer may also incorporate a DFB ICL seed laser, emitting at a single frequency near the center of the comb gain spectrum, to lock the frequencies of the two low-noise combs. For example, it could reside between the two combs illustrated in Figure 12, with both ends of its curved cavity also terminating at the HR-coated facet. 

## 6. Practical Considerations

In general, an ICL incorporated into a PIC should perform quite similarly to stand-alone devices (e.g., see [18]) that are configured and processed similarly. Our simulations indicate that, even when the layer design is modified to make the bottom SCL thicker and the top SCL thinner, to promote efficient out-coupling to a passive waveguide, the effect on laser performance is relatively modest. For most of the configurations discussed above, an HR-coated facet defines at least one end of the cavity as in a conventional ICL. If butt-coupling to a passive waveguide defines the other end and no additional elements are integrated into the cavity, the mirror loss and laser operation are quite analogous to those of standard ICLs with output mirrors defined by uncoated or AR-coated facets. If both ends are defined by HR-coated facets, as in Figure 8, the threshold current density and drive power are likely to be lower due to the lower mirror loss. ICLs with a sensing waveguide incorporated into the cavity will experience a trade-off between lower sensitivity when the sensing section is short, against higher loss and lower laser efficiency when it is lengthened. Similarly, the designs that couple light laterally to an output waveguide must trade greater isolation from potential external feedback when the out-coupling per pass is weak, against higher laser efficiency when it is stronger. 

It was mentioned above that the mode overlap, for coupling the beam propagating in a passive sensing waveguide to an ambient sample gas, is relatively weak. Therefore, it may be advantageous to structure the waveguide so as to provide more coupling than the simple top hats of Figure 2. For example, a slot with sub-wavelength dimensions along the lateral (stronger TE coupling) [52] or vertical (stronger TM coupling) [53] waveguide axis can substantially enhance the modal overlap. A related approach is to pattern a subwavelength-scale grating along the longitudinal axis [54,55], which, besides enhancing the coupling, also slows the light propagation at frequencies near the band edge of the 1D photonic crystal. Of course, such structures can be challenging to process in III-V materials (a potential advantage of Si PICs), even at the longer mid-IR dimensions, and the additional loss due to scattering by sidewall nonuniformities may potentially outweigh the theoretical advantages [56]. Plasmonic nanostructures and photonic crystals can also enhance the coupling to ambient [57,58], with surface plasmon modes in a heavily doped semiconductor [59] possibly providing lower mid-IR absorption loss than a plasmonic structure patterned into a deposited metal. A photonic crystal silicon waveguide was recently reported to yield the sub-parts-per-million detection of ethanol at λ = 3.47 µm, although in that demonstration the ICL source and InSb detector resided off-chip [60].

It may also be possible to enhance the detection sensitivity by coating the top and sides of the waveguide with a sorbent that selectively interacts with and concentrates a given class of chemicals, which diffuse into the sorbent layer. This can increase the molecular concentration, and hence the detection sensitivity to a given chemical by orders of magnitude [61,62,63]. 

A further alternative is to incorporate a free space sensing area into the PIC, which provides full modal overlap with the sample gas. This approach has the obvious disadvantage of weak coupling into the detector due to diffraction following free space propagation over any appreciable path length. A possible solution is to incorporate a hollow-core waveguide that provides flow of the sample gas [64]. While single-mode propagation may be challenging to realize in practice, the hollow waveguide should nonetheless trap and direct the beam over some extended distance before losses substantially dissipate its intensity. 

Note that the PIC can spectroscopically probe a narrow analyte fingerprint absorption line in multiple ways. These include tuning the wavelength of a single-mode laser source (most commonly by current or temperature, although a broader range may be attainable with a sampled grating [65] or related configuration), tuning the resonance of a resonator sensing waveguide, such as a ring (by temperature), tuning the wavelength of an in-plane resonant cavity detector (by temperature), separating the spectral components of a broadband signal (e.g., using an AWG), integrating multiple sensors operating at different absorption and reference wavelengths on the same chip, or dual-comb spectroscopy with multi-heterodyne detection. The latter may ultimately offer the most attractive combination of broad spectral bandwidth, short acquisition time, and low drive power consumption. Note also that, when more than one of these methods provides a narrow or comb-like spectral response (e.g., a single mode laser combined with a ring resonator sensor and/or resonant cavity detector), the multiple resonance wavelengths must be matched by careful design and calibration, or independent tuning (probably with temperature) of the different components. 

We emphasize again that, while ICL-based PICs integrated on the native III-V substrate were assumed as the default in most of the preceding discussions and analyses, all of the same architectures may also be applied to QCLs for the coverage of spectral bands extending far beyond the mid-IR (i.e., LWIR out to THz). A primary difference, however, is that thermal management becomes much more critical. QCLs require much more input power just to reach threshold, whereas even an epitaxial-side-up mounted ICL can often operate in cw mode. Additionally, whereas integration on silicon rather than the native GaSb or InP substrate requires additional processing steps, it can offer more mature processing of gratings and high-resolution nanoscale structures, along with the potential to combine sensors operating in multiple spectral bands on the same chip [22,23,24].

## 7. Conclusions

The preceding sections have presented a framework for constructing ICL-based PICs on the native GaSb substrate. Following the redistribution of the separate confinement layer thicknesses to make the bottom SCL thicker, the same MBE-grown wafer material becomes suitable for integrating interband cascade lasers, interband cascade detectors, and connecting passive waveguides on the same chip. For chemical sensing, the tops and sides of the passive waveguides can be exposed to ambient to provide evanescent coupling to a sample gas.

Some configurations incorporate the sensing waveguide into the laser, which allows both ends of the cavity to be defined by HR-coated facets for low mirror loss and isolation from external feedback. Absorption by the analyte can then be detected by monitoring the laser’s compliance voltage, or by incorporating an ICD into the same cavity.

We also described a laser configuration that extracts light by lateral evanescently coupling to a passive waveguide. This again allows both ends of the laser cavity to be defined by HR-coated facets and provides the incremental adjustment of the coupling efficiency per pass. Stronger coupling gives higher output power and wall-plug efficiency, whereas weaker coupling provides optimal laser stability when relatively modest power is required for sensing. It is well known that fluctuations associated with external optical feedback can be a major source of instability in conventional edge-emitting semiconductor lasers. A further advantage of the lateral power extraction is that both ends of the laser cavity can be terminated at the same HR-coated facet. This adds considerable flexibility to the PIC layout, which can, e.g., group one or more lasers at one end of the chip, which may be mounted epitaxial-side-down, while one or more sensing waveguides are exposed to ambient at the other end. Laser cavities with lateral extraction and termination on both ends by HR-coated facets may also operate at lower drive powers than has been possible up to now, since a very short cavity would no longer induce excessive mirror loss. 

We finally discussed configurations that integrate a laterally-out-coupled ICL, passive sensing waveguide, and ICD on the same PIC. The ICD’s sensitivity specific detectivity D* may be enhanced by incorporating it into a resonant cavity defined by DBRs. A particularly promising prospect is dual-comb spectroscopy on an ultra-compact chip, with broad and rapid spectral coverage, combined with low drive power.

Assuming that mature designs and high-yield processing procedures can be developed, chemical detection PICs will be suitable for mass-producing hundreds of devices on the same chip in a standard cleanroom. Reference [66] reviews the relatively advanced status of InP-based photonic integrated circuits operating at shorter wavelengths in the near IR, which can now combine numerous optical elements on a chip. Although GaSb-based PICs and GaSb-based device fabrication, in general, are much less mature [67], the simplest on-chip sensing configurations described above do not require methods especially more challenging than those already used routinely to process shortwave-IR and mid-IR lasers and detectors on GaSb. After patterning a large number of PICs on a die, individual sensors can be singulated to form a package that is both extremely compact and inexpensive. Lasers, detectors, and passive waveguides can also be integrated into more complex PICs that incorporate other components for enhanced capabilities, such as the multi-heterodyne detection of an off-chip signal [68]. While the relevant fabrication protocols have yet to be developed, they appear quite feasible as an extension of current capabilities. 

Since interband cascade photovoltaic devices have been demonstrated, there is even the potential for integrating a solar cell power source on the same PIC with the light source, passive sensing waveguide, and ICD, to provide an even more complete stand-alone chemical detection package. While such a power source is unlikely to provide enough current to reach the ICL lasing threshold, it may at least be sufficient to drive a broadband ICLED.

## Data Availability

The data presented in this study are available on request from the corresponding author, subject to approval by the Naval Research Laboratory.
